# Short-Term Effects of Deliberate Subparaneural or Subepineural Injections With Saline Solution or Bupivacaine 0.75% in the Sciatic Nerve of Rabbits

**DOI:** 10.3389/fvets.2020.00217

**Published:** 2020-05-12

**Authors:** Francisco G. Laredo, Eliseo Belda, Marta Soler, Francisco Gil, José Murciano, Joaquín Sánchez-Campillo, Amalia Agut

**Affiliations:** ^1^Department of Medicine and Animal Surgery, Faculty of Veterinary Science, University of Murcia, Murcia, Spain; ^2^Department of Comparative Anatomy and Pathological Anatomy, Faculty of Veterinary Science, University of Murcia, Murcia, Spain

**Keywords:** intraneural puncture, intrafascicular, extrafascicular, sciatic nerve, rabbit

## Abstract

**Background:** Ultrasound (US)-guided techniques for peripheral nerve blockade have revealed that intraneural injections are relatively frequent and not necessarily associated with neurological deficits.

**Objectives:** To evaluate the short-term effects of deliberate injections performed under direct vision in two different sites of the sciatic nerve (ScN).

**Material and Methods:** Seventy-two New Zealand white rabbits randomly assigned to one of four experimental groups (*n* = 18) were employed. All procedures were conducted at a proximal femoral level where the ScN incorporates the common peroneal nerve and the tibial nerve (TN). Fixed volumes of 0.5 ml of saline solution (ES group) or bupivacaine 0.75% (EB group) were administered extrafascicularly inside the paraneurium of the ScN or intrafascicularly (IS and IB groups) under the epineurium of the TN. Cross-sectional area (CSA) and relative echogenicity (RE) of the entire ScN were determined by US before injections, after injections, and at 3 and 7 days. ScN samples were obtained for structural and ultrastructural histopathological studies. Proprioceptive, sensorial, and motor function were clinically evaluated on a daily basis.

**Results:** The CSA of the ScN increased significantly immediately after injections when compared with pre-injection values in all groups (*p* < 0.05). The RE of the ScN decreased in relation to pre-injection values in all groups (*p* < 0.05). The CSA and RE of the ScN returned to normal values 7 days after injections in almost all groups. Injected nerves showed histological signs of mild perineural inflammation. Histopathological scores were not significantly different between groups (*p* > 0.05). The architecture of the ScN was preserved in all rabbits at 3 days and in 31/32 rabbits at 7 days. A focal area of damaged nerve fibers with degeneration of the axons and myelin sheath affecting the TN was observed in one rabbit of the IB group. Nerve function was not clinically impaired in any case.

**Conclusion:** Despite the lack of severe nerve disruption observed in most rabbits, the evidence of a focal area of damaged nerve fibers in one rabbit injected intrafascicularly with bupivacaine confirms that intrafascicular injections should be avoided as they may increase the risk of nerve damage.

## Introduction

Ultrasound-guided sciatic nerve (ScN) blocks are used for a variety of surgical procedures to reduce the need for general anesthesia and its associated risks (1, 2). A complication of peripheral nerve blocks is the occurrence of iatrogenic nerve damage, but the incidence of long-term neurologic injuries is relatively low (3–6). US-guided techniques for regional anesthesia have shown that peripheral nerves injected with local anesthetic do not inevitably suffer permanent neurological injuries (7–15), and some authors, contrary to other reports (16–19), have challenged the risk of intraneural injections (7).

**Figure 1:**
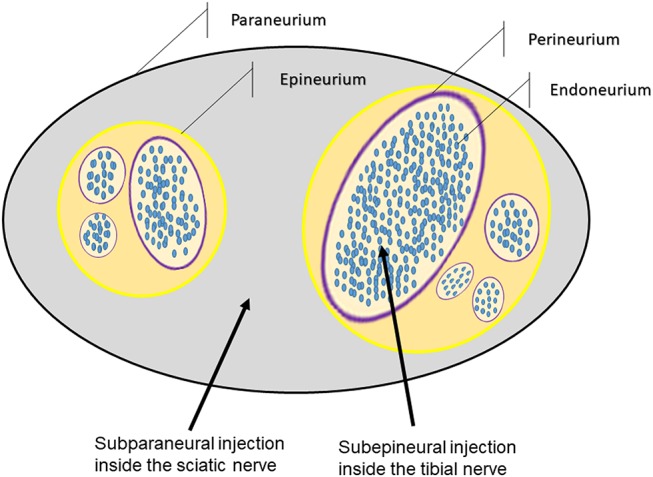
Diagram of the structure of the sciatic nerve in rabbits showing the sites of injections.

The ScN consists of the tibial (TN) and common peroneal (CPN) nerves (Figure 1) surrounded by a common connective sheath, named paraneurium (circumneurium) (20–23). Studies conducted in a variety of species, including human beings, documented that administration of local anesthetics into this nerve may not affect the integrity of nerve fascicles (9, 12, 14, 21, 22, 24). These results should be interpreted with caution because the precise sites of the intraneural injection are not adequately described in most of these studies, and injections made within the paraneurium but outside the epineurium of the TN or CPN should not be really considered as intraneural (22, 23, 25–27). There are also important microanatomical variations in the structure of different nerves within the same species, and of the same nerves between different species (25, 26), which may produce different outcomes when translating these findings into clinical practice.

The objective of the present study was to assess the short-term effects of deliberate intraneural injections of saline solution or bupivacaine beyond the epineurium of the TN in rabbits, compared to their extrafascicular administration in the paraneurium of the ScN, correlating the ultrasonographic findings after the injections with alterations of the nerve structure and neurological function. We hypothesized that the intraneural administration of bupivacaine 0.75% inside the TN would produce the most intense disruption in the nerve structure and function. A second objective was to describe the anatomical and sonoanatomical characteristics of the ScN in rabbits.

## Materials and Methods

This research was designed as a prospective, randomized, blinded, experimental study, and it was performed in accordance with the guidelines for animal research and the 3R principles of the EU directive. It was approved by the University of Murcia Ethics Committee (approval number 218/2016) and followed the animal research reporting of *in vivo* experiments (ARRIVE) guidelines. Seventy-two purpose-bred New Zealand white rabbits purchased from Granja San Bernardo (Navarra, Spain), males (*n* = 60) and females (*n* = 12), with a mean age of 15.2 ± 1.6 weeks and a mean body weight of 3.31 ± 0.41 kg were employed. Animals were healthy based on physical examination and free of proprioceptive, sensorial, or motor deficits in the hind limbs. Rabbits were individually caged in a room provided with controlled temperature (22 ± 3°C) and light (12 h). Animals were fed with pelleted concentrated feed with free access to food and water throughout the acclimatization and study period. An acclimatization period of at least 1 week was observed before the experiments. On the day of the trials, food was withheld for at least 6 h, but rabbits were allowed free access to water. All procedures started at 9.00 a.m.

**Figure 2:**
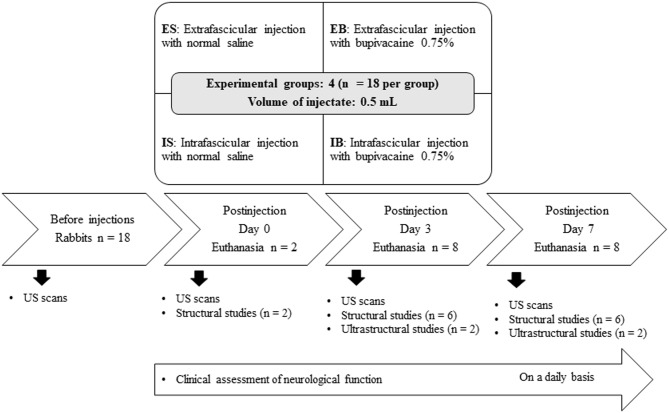
Experimental groups and timeline schematic diagram of the experimental design.

Rabbits were divided by gender and each subpopulation was randomly assigned (by lottery) to one of four experimental groups of 18 animals each (15 males and 3 females per group). In accordance to the experimental group, rabbits received 0.5 ml of saline solution (ES group) or bupivacaine 0.75% (EB group) extrafascicularly inside the paraneurium of the ScN, or intrafascicularly under the epineurium of the TN (IS and IB groups) (Figures 1, 2). Anesthesia was achieved with ketamine 25 mg/kg (Aneskine, Dechra SLU, Barcelona, Spain), medetomidine 50 μg/kg (Domtor, Ecuphar veterinaria SLU, Barcelona, Spain), and buprenorphine 0.02 mg/kg (Buprenodale, Dechra Limited, Staffordshire, United Kingdom) administered subcutaneously (SC) in the neck area. A 24-G catheter was aseptically placed in the marginal auricular vein, and a balanced solution of crystalloid fluid (Lactato de Ringer, B. Braun Vetcare SA, Rubi, Spain) was administered at a rate of 4 ml/kg/h. Animals were connected via a fitted face mask to an Ayre's T-piece breathing system to receive supplementary oxygen (100%) and, if necessary, isoflurane 2.5% (Isoflo, Ecuphar veterinaria SLU, Barcelona, Spain).

Images with identical imaging variables than the studied nerves were obtained from three soft-tissue equivalent ultrasonographic phantoms for the US study. The phantoms were made and stored in sterile blood transfusion pockets as previously described (14, 28). The hair from the sacroiliac region to just below the stifle on the dorsal and lateral aspects of the left and right limbs of the rabbits was clipped. The skin was cleaned and coupling gel applied with the animals positioned in the right lateral position. A multifrequency (4–13 MHz) linear transducer (MyLab 70, Esaote Biomedica, Genoa, Italy) was employed for the ultrasound examinations. The same investigator (AA) carried out all the US scans. Constant focus, brightness, contrast, and gain settings were used in all the scans. US images obtained from the three phantoms, as well as from the studied nerves before the procedures, immediately after the injections, and at 3 and 7 days were digitally recorded. Later, the recorded images were analyzed by the same investigator (MS) using an image-analysis system (Microm Image Processing software) to measure the cross-sectional area [CSA (mm^2^)] and relative echogenicity (RE) of the entire ScN using a scale of 256 gray levels (0 = black; 255 = white) as previously described (13). Three different CSA and RE measurements from each image were recorded, and then, the mean value of these measurements was considered as the CSA or RE value.

The ScN was imaged along the lateral surface of the thigh from proximal just at the level of the greater trochanter to the popliteal region near the stifle to standardize the examinations (proximal, mid-femoral, and popliteal approaches). The transducer was placed in the transverse plane just distal and caudal to the greater trochanter and then directed toward the distal aspect, close to its origin to the point where the two nerve components of the ScN, the CPN and the TN, were clearly identified under the paraneurium. The mark of the transducer in longitudinal and transverse planes was positioned in a proximal and cranial direction, respectively. Longitudinal images of the ScN were obtained by rotating the transducer 90° clockwise from the position used to obtain the transverse images. A proximal approach to the ScN, at the first third of the femur, was selected to standardize all procedures. This approach allowed reaching precisely the subparaneural space of the ScN or the subepineural space of the TN accordingly to the experimental group.

Once the pre-injection US scans were completed, the ScN was approached on the right limb through a lateral incision, which was extended proximally to the greater trochanter and distally to the mid-third of the femur. The same investigator (JM) undertook the surgical exposition of the ScN in all cases. The ScN was identified after retraction of the biceps femoris caudally and of the vastus lateralis muscle cranially, which exposed the nerve in an adequate length without further manipulations. Long bevel 0.5 × 40 mm, 25-G, 114″ hypodermic needles (Sterican, B. Braun AG, Melsungen, Germany) connected to a 1-ml syringe were employed to perform the injections. Injection pressures (psi) were recorded by an electronic manometer (Manometer PCE-917, PCE Ibérica, Albacete, Spain) connected through a three-way stopcock and a low compliance plastic tube to the system. The system was prefilled with saline solution (Suero fisiológico salino, B. Braun Vetcare SA, Rubi, Spain) or bupivacaine (Bupivacaine 0.75%, B. Braun Vetcare SA, Rubi, Spain) accordingly to the experimental group. Extrafascicular injections were made within the paraneurium of the ScN, whereas intraneural injections were performed under the epineurium of the TN. The needles were inserted under direct vision, along the long axis of the ScN and with an approximate angle of 30°, by the same investigator (FL) with the help of surgical loupes. This investigator was the only one aware of the intervention protocols. Once the tip of the needle was located in the appropriate site, the solution of injectate was administered over 25 s (1.2 ml/min).

The same investigator (EB) evaluated the presence of neurologic signs in all the rabbits on a daily basis. The leg position, proprioception, standing, and walking patterns were evaluated, firstly, by observation of the rabbits undisturbed and standing, and later at walking after they were gently stimulated. Sensory function was also assessed by pinching the dermatomes innervated by the CPN and TN (dorsal and plantar skin areas) of the injected hind limb with forceps. The forceps was progressively closed for a maximum time of 10 s until a pain-related response was noted or until the first ratchet notch was locked. This investigator also assessed the general status of the rabbits three times daily, observing the intake of food and water and searching for signs of gastrointestinal dysfunction, and behavioral and postural indicators of pain in rabbits (29). This evaluation was completed by observing the response of the animals to firm application of digital pressure around the wound area. Pain was graded as absent, mild (rabbits exhibiting fewer than two pain-related indicators), moderate (evidence of three to five pain-related indicators), and severe (evidence of six or more pain-related indicators). Buprenorphine 0.02 mg/kg (Buprenodale, Dechra Limited, Staffordshire, United Kingdom) was readily available to be administered SC during the early postoperative period if it was necessary.

Rabbits were humanely euthanized at the scheduled observational times (Figure 2). Animals were anesthetized as described above, and then a volume of 3–5 ml of pentobarbital 400 mg/ml (Euthasol, Ecuphar veterinaria SLU, Barcelona, Spain) was administered intravenously through a 24-G catheter placed in an intact marginal auricular vein. Nerves were carefully dissected to obtain samples with a length of 1 cm (0.5 cm on either side of the injection sites). Nerve samples were fixed in 10% neutral buffered formalin for at least 72 h. Tissue blocks were processed for paraffin embedding and cross-sections (8-μm thicknesses) stained using H&E, Luxol fast blue, and Masson's trichrome. For each nerve sample, at least five consecutive sections separated by 100 μm were examined to search for histological evidence of nerve inflammation and injury. These sections were photographed by an image analysis device (Sigma-Scan Pro. 5.0, Jandel Corp., Sausalito, Cal, USA) connected to a light photomicroscope (Leitz Dialux 20, E. Leitz KG, Hamburg, Germany). Evidence of nerve inflammation was defined as the presence of inflammatory cells around vessels and fascicles of the injected nerves as previously reported (28). Evidence of nerve injury was defined as the loss of integrity in the perineurium (13) with signs of myelin damage (30). The presence of neural inflammation and injury was graded on a four-point nominal score based on previous descriptions (11, 30) as follows: 1 = no presence inflammatory cells, 2 = areas with slight accumulation of inflammatory cells, 3 = areas with abundant accumulation of inflammatory cells, and 4 = signs of structural nerve injury. In addition, nerve samples were taken for ultrastructural studies with transmission electron microscope (TEM). Nerve samples were obtained from pieces of 1 mm thick ×2 mm longitudinal segments, which were fixed in 2.5% glutaraldehyde in buffered 0.1 M cacodylate (pH 7.2–7.4) for 3 h at 4°C. Additional TEM processing was performed in the Microscopy Core Facility of our institution according to the standard protocol for epoxy embedding. Transverse semithin sections (5 μm thick) were obtained with an ultramicrotome (Leica Ultracut UCT-UC6, Heidelberg, Germany) and stained with Toluidine Blue for being observed by light microscopy. Ultrathin sections (70 nm) were also obtained from all blocks with a Leica ultracut ultramicrotome. These samples were stained with aqueous saturated uranyl acetate and Reynolds lead citrate and viewed using a digital TEM (JEOL, JEM/1011 model, Tokyo, Japan) at 80 kV. The same investigators (FG and JS-C) carried out all the histopathological studies.

Additionally, six rabbits from the experimental groups were randomly selected to be used as negative controls. In these animals, the left ScN was surgically exposed. The histological images obtained from the left ScN of these animals were employed to assess the normal characteristics of this nerve in rabbits and for detecting potential histological changes caused by the surgical exposure of the target nerves. Images from these nerves were also used to quantify histologically the CSA of the TN (mm^2^).

Assuming that the incidence of histological structural nerve injury would be ~50% in the IB group and 5% in the ES group (11, 17), a sample size calculation estimated that 14 animals per group were required (β = 0.10, alpha = 0.05). This number was increased to 18 rabbits per group to account for potential dropouts. Statistical tests were performed using IBM SPSS statistics 24.0 (IBM Spain, Madrid). Normally distributed data (established by Shapiro–Wilk test) are expressed as mean ± standard deviation (X ± SD). Comparisons between groups were carried out using a one-way ANOVA with Tukey *post hoc* analysis. Comparisons intragroup for the different observational times were carried out using ANOVA for repetitive measurements with Bonferroni *post hoc* analysis. Non-parametric data, expressed as median ± range, were compared using a Fisher's exact test. Statistical significance was defined as *p* < 0.05.

## Results

Recovery from procedures was uneventful, and vital signs and body temperature were assessed until rabbits made a full recovery from anesthesia. All rabbits completed this research without signs of discomfort, pain, or neurological dysfunction in any case. Buprenorphine was administered every 8 h to animals that exhibited a reduction in food or water intake plus two or more behavioral or postural indicators of pain. Signs compatible with moderate pain were detected in nine rabbits during the first 24 h after the injections. These animals received buprenorphine and gradually recovered a normal behavior.

**Figure 3:**
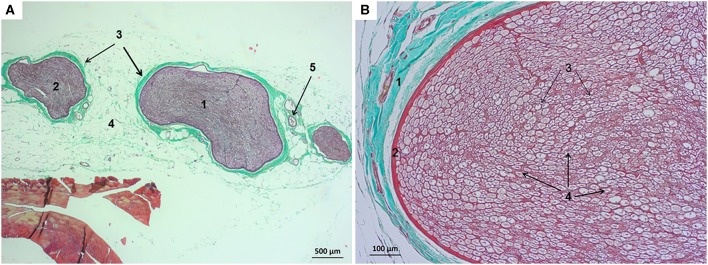
Structure of the sciatic nerve in the rabbit. **(A)** Cross-sectional image stained with Masson's trichrome (magnification initially: 4×): (1) tibial nerve, (2) common peroneal nerve, (3) epineurium, (4) paraneurium or common connective sheath, and (5) blood vessels. **(B)** Cross-sectional image of the tibial nerve stained with Masson's trichrome (magnification initially: 20×): (1) epineurium with small blood vessels, (2) perineurium, (3) endoneurium, and (4) nerve fibers.

The ScN was located between the muscles of the thigh lying medial to the biceps femoris and caudal to the femur. In the area where procedures were carried out at the first third of the femur, this nerve was oligofascicular and composed of the CPN and the TN. These two nerves were compact and monofascicular and mainly integrated by neural tissue (Figure 3). The TN was located caudal to the CPN and had a greater CSA. The average CSA of the TN determined from the histological negative control samples was of 0.607 ± 0.060 mm^2^ (range: 0.500–0.717). The two nerves were surrounded by an individual thin perineurium, which was closely adhered to a thicker epineurium where a few small blood vessels were identified. The CPN and TN were surrounded by a common thick paraneural compartment, which was rich in adipose and connective tissue where larger blood vessels were evidenced (Figure 3).

**Figure 4:**
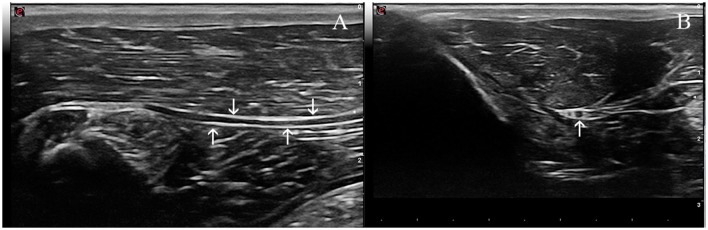
Ultrasound images of the sciatic nerve in the rabbit. **(A)** Longitudinal ultrasound image of this nerve (arrows), which appears as a tubular hypoechoic structure, outlined by hyperechoic lines (arrows). **(B)** Transverse ultrasound image of the sciatic nerve. The two components of this nerve are readily distinguished and appear as two ovoid hypoechoic structures surrounded by a thin hyperechoic rim; the smaller and more cranial one representing the common peroneal nerve, and the larger and more caudal the tibial nerve (arrow).

The ScN was easily visualized by ultrasound in all the rabbits. It was observed as a double hypoechoic rounded structure (common peroneal and tibial nerves) surrounded by a hyperechogenic rim, which was the external common connective sheath. In longitudinal views, the ScN appeared to be as a tubular hypoechogenic structure delimited by two hyperechoic lines (Figure 4).

Injections were completed without incidents in all cases, but produced discrete areas of focal hemorrhage in the paraneural tissues. Subparaneural injections produced an accumulation of fluid with the form of a bulla in an approximate length of 4–5 mm. In some cases, part of the injected solution dripped out once the needle was withdrawn from the nerve. Intraneural injections inside the TN produced an accumulation of fluid with a fusiform shape in an approximate length of 2–3 mm. In all cases, part of the injected solution was ejected from the TN once the needle was withdrawn, due to the rapid retraction of the TN. Bupivacaine injections resulted in sensory and motor blockade for up to 16 h. Contrarily, animals exhibited a normal sensory and motor function after injections with saline in all cases.

**Figure 5:**
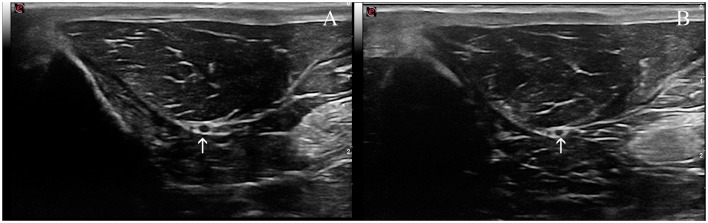
Transverse ultrasound images of the sciatic nerve. **(A)** Three days and **(B)** seven days after the subperineural injection of bupivacaine in the tibial nerve (arrow). Note that the cross-sectional area of the tibial nerve is greater on the image from 3 days than 7 days. The changes of nerve echogenicity at these days were unremarkable.

CSA values of the ScN determined by US immediately after injections significantly increased when compared with pre-injection values in all groups (*p* = 0.001 in ES and IB groups, *p* = 0.002 in EB group, *p* = 0.008 in IS group) (Table 1). RE values decreased in relation to the pre-injection values in all groups (*p* = 0.004 in ES and IS groups, *p* = 0.002 in EB group, *p* = 0.001 in IB). CSA and RE values returned to basal (pre-injection) values in almost all groups 7 days after injections (Figure 5). Apart from these findings, US scans of the ScN were normal during the study period. Injection pressures never exceeded 20 psi (138 kPa) in any case. Mean maximum injection pressures were significantly lower in the ES and EB compared to the IS and IB groups (*p* = 0.001) (Table 2).

**Table 1:** Mean (±SD) cross-sectional area (CSA) and relative echogenicity (RE) of the sciatic nerve in rabbits before and after the administration of 0.5 ml injectate (S, saline or B, bupivacaine) extrafascicularly (E) inside the sciatic nerve or intrafascicularly (I) inside the tibial nerve.

	**Group**	**Before injection**	**Day 0 after injection**	**Day 3 after injection**	**Day 7 after injection**	***P*-value**
CSA (mm^2^)	ES	1.45 ± 0.13^a^	1.74 ± 0.10^b^	1.46 ± 0.03^a^	1.43 ± 0.04^a^	<0.001
	EB	1.43 ± 0.10^a^	1.89 ± 0.27^b^	1.53 ± 0.10^a^	1.48 ± 0.10^a^	0.002
	IS	1.32 ± 0.04^a^	1.78 ± 0.30^b^	1.50 ± 0.09^b^	1.37 ± 0.06^a^	0.008
	IB	1.43 ± 0.09^a^	1.95 ± 0.15^b^	1.57 ± 0.13^c^	1.38 ± 0.05^a^	<0.001
RE (gray levels)	ES	46.06 ± 12.71^a^	21.19 ± 8.20^b^	32.03 ± 15.52^c^	44.06 ± 10.10^a^	0.004
	EB	41.09 ± 16.61^a^	19.68 ± 9.07^b^	25.65 ± 13.40^c^^,^ ^d^	30.35 ± 11.40^d^	0.002
	IS	34.82 ± 21.44^a^	14.96 ± 6.87^b^	26.66 ± 9.48^c^	32.55 ± 15.11^a^^,^ ^c^	0.004
	IB	50.30 ± 14.94^a^	21.03 ± 11.04^b^	32.93 ± 5.86^c^	40.16 ± 7.25^a^	<0.001

a, b, c, d *Values with a different superscript within a row differ significantly*.

**Table 2:** Mean (±SD) maximum values of pressure (psi) during the administration of 0.5 ml of injectate (S, saline or B, bupivacaine) extrafascicularly (E) inside the sciatic nerve or intrafascicularly (I) inside the tibial nerve.

**Group**	**Pressure**	***P*-value**
ES	3.80 ± 1.79^a^	
EB	6.25 ± 3.05^a^	0.085
IS	14.13 ± 4.61^b^	
IB	13.25 ± 6.06^b^	0.60

a, b *Values with a different superscript within a column differ significantly (P < 0.001)*.

**Figure 6:**
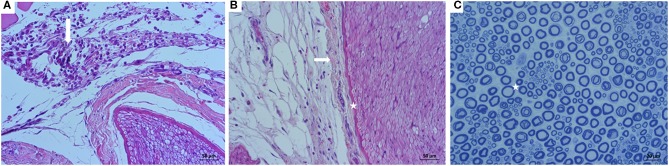
Images of the sciatic nerve after subepineural injections with bupivacaine in the tibial nerve. **(A)** Cross-sectional view of a nerve stained with H&E (magnification initially: 20×) 3 days after the injection: perineural areas with slight accumulation of inflammatory cells are observed (arrow). **(B)** Cross-sectional view of another nerve stained with H&E (magnification initially: 20×) 3 days after injection: no changes in the integrity of the epineurium (arrow) or perineurium (star) are detected. **(C)** Transverse semithin section of a tibial nerve stained with toluidine blue (magnification initially: 40×) 7 days after injection: a normal structure and distribution of the axons and myelin sheaths is observed (star).

**Figure 7:**
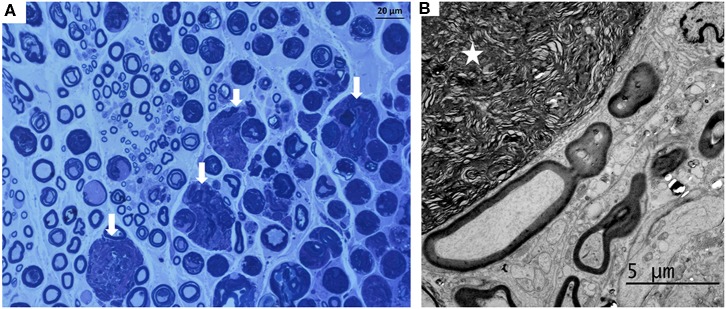
Images of the sciatic nerve 7 days after a subepineural injection with bupivacaine in the tibial nerve. **(A)** Transverse semithin section stained with toluidine blue (magnification initially: 40×): a local area with degenerated myelin fibers (arrows) was evidenced in one rabbit. **(B)** Electron microphotography (magnification initially: 5,000×) of the tibial nerve of this rabbit showing degenerated myelin (star).

The histological study performed on the ScN negative controls did not evidence signs of neural inflammation or structural disruption due to surgical manipulation in any case. Contrarily, injected nerves showed histological signs of mild perineural inflammation characterized by slight accumulation of inflammatory cells in the four groups (Figure 6). Median (range) histopathological scores were not significantly different between the experimental groups (*p* > 0.05) (Table 3). The subparaneural (extrafascicular) injection with saline or bupivacaine did not produce histological signs of nerve fiber damage, as the internal architecture of the nerve fascicles revealed a normal structure and distribution of the axons and myelin sheath in all cases. Similar findings were observed histologically after the subepineural (intrafascicular) injection with saline and bupivacaine (Figure 6) in 31/32 rabbits. However, in one rabbit of the IB group, a focal area of damaged nerve fibers with degeneration of the axons and breakdown of the myelin sheath was observed 7 days after the injection (Figure 7). In spite of these findings, no clinical evidence of neurological deficits was observed.

**Table 3:** Median values (range) of the histological scores observed after the administration of 0.5 ml injectate (S, saline or B, bupivacaine) extrafascicularly (E) inside the sciatic nerve or intrafascicularly (I) inside the tibial nerve.

**Experimental group**	**Histopathological scores**		
	**Day 0 after injection**	**Day 3 after injection**	**Day 7 after injection**
ES	1 (0)	1.5 (1)	1 (0)
EB	2 (0)	2 (1)	1.5 (1)
IS	1 (0)	1.5 (1)	2 (0)
IB	1 (0)	2 (1)	2 (2)
*P*-value	0.446	0.754	0.323

## Discussion

The administration of fixed volumes of 0.5 ml of saline or bupivacaine 0.75% solutions extrafascicularly in the ScN (subparaneural injections) or intrafascicularly inside the TN (subepineural injection) produced a significant increase in the CSA and a significant decrease in the RE of the ScN evidenced by US immediately after injections. Histological changes after injections were mild in most rabbits, and motor, proprioceptive, and sensitive functions were not clinically impaired in any case during the study period. However, a focal area of damaged nerve fibers with degeneration of the axons and myelin sheath affecting the TN was observed in one rabbit of the IB group, indicating that intraneural injections may increase the risk of structural nerve damage particularly if bupivacaine is administered.

Intraneural injections have been classically identified as one of the main risks of nerve injury after PNB techniques (3–6, 16–19, 31). However, previous descriptions have documented that the intraneural administration of local anesthetics into the ScN may not affect the integrity of nerve fascicles (7, 9–12, 14, 15, 22). These contradictory reports could be explained by differences in the terminology employed by previous authors to define intraneural injections (23, 25–27), as the precise sites of injection are not clearly indicated in most studies (3, 7, 9–11, 14). Considering the anatomical structure of the ScN, injections made within its paraneurium but outside the epineurium of the TN or CPN should not be considered as “truly” intraneural (22, 23, 25–27). It should also be considered that US-guided techniques do not offer the resolution needed to differentiate extrafascicular from intrafascicular injections (6, 10, 22, 23, 25, 26). For the above reasons, intraneural injection sites were clearly defined and procedures were carried out under direct vision in this research.

Our results showed that the ScN is oligofascicular in rabbits as previously described (32). It is composed by the CPN and TN, which were compact, monofascicular, and mainly integrated by neural tissue. These nerves were surrounded individually by a thin perineurium closely adhered to a thicker epineurium. Therefore, the injections performed during this research inside the TN were in fact subperineural injections. The CPN and TN were also surrounded in rabbits by a common thick paraneurium or subcircumneural compartment similarly to the ScN of human beings (20–23). It has been reported that the ScN in rabbits has a median of seven fascicles with a median CSA of only 0.095 mm^2^, whereas in humans, it has a median of 58 fascicles with a median CSA of 0.174 mm^2^ (32). The huge differences in the nerve architecture, nerve size, and fascicles size between rabbits and humans could limit the validity of translational studies carried out in rabbits on the effects of intraneural injections (32). Contrary to our study, these authors performed the anatomical description of the ScN in rabbits at the popliteal region. Our research found that the ScN in rabbits had only three fascicles at the injections site and that the TN consisted of only one big fascicle. It is known that the ScN has important anatomical differences between its proximal and distal regions (33), which could explain these discrepancies, as all procedures were carried out in our study at the first third of the femur, just below the level of the greater trochanter.

The extraneural injections produced a subparaneural bulla that spread outside the main nerves, whereas the intraneural injections produced a fusiform accumulation of fluid along the TN. The TN moved slightly away from the tip of the needle in some cases, without impairing the completion of the intrafascicular injections. Mean maximum injection pressures (psi) were significantly lower during extraneural injections compared to intraneural injections. These values never exceeded a dangerously high level probably due to the small volume of the injections and the slow rate of administration. It has been reported that a high injection pressure (≥25 psi) may predict histological and functional nerve injury after intraneural injection (16). Nerves are not homogeneous unitary structures; therefore, it could be possible to inject a fluid beyond the epineurium without noticing a dramatic increase in the injection pressure (14). Some false positives were detected in or study during extrafascicular injections, which were probably caused by a transient occlusion of the tip of the needle. These findings reflect the low specificity of pressure monitoring devices to detect intraneural injections, as previously described by others (34).

The CSA of the ScN increased significantly after injections, compared with the pre-injection values. Contrarily, the RE values of the ScN decreased significantly after the injections without observing clinical evidences of neurological deficits during the study period considered here. These findings support previous research regarding the fact that modifications of the echogenicity (RE) or size (CSA) of peripheral nerves due to intraneural injections were not associated with deficits of clinical relevance in proprioceptive or motor functions (8, 10, 11, 14, 15). Histopathological scores were similar in the four experimental groups, and most nerves showed histological signs of mild perineural inflammation, without changes in the integrity of epineurium and perineurium. Post-traumatic inflammation rather than structural damage has been described as the more common consequence of peripheral nerve perforation (30) or injection (8, 10, 11, 14). The subparaneural injection with saline or bupivacaine solutions inside the ScN did not result in nerve fiber damage at the observational times considered in our research. No other clinical evidence of neurological deficits or damage was observed in the paraneural injection groups. These findings may support the practice of subparaneural injections inside the ScN in a clinical setting. It has been reported that these injections may provide the operator with the opportunity to inject between the CPN and TN without injuring the epineurium of any individual nerve. This could be useful to achieve a fast onset of action and a highly successful block at lower doses of local anesthetic (15, 27).

Contrary to our hypothesis, histological signs of mild perineural inflammation, without disruption of the epineurium and perineurium, were also observed after the intrafascicular injections inside the TN. However, one rabbit of the IB group showed a local area of damaged fibers with degeneration of the axons and breakdown of myelin sheath at 7 days. No other clinical evidence of neurological deficits or damage was observed in the intrafascicular injection groups. As the TN in rabbits is mainly composed of neural tissue, it may produce a tight, solid, and low compliance structure, which may reduce the risk of axonal disruption after intrafascicular injections inside this nerve. In a recent human cadaveric study, the administration of 20 ml of diluted heparinized blood in the TN did not disrupt the perineurium nor other neural structure, similar to our observations (22). Our results support previous descriptions documenting that severe neurologic injuries are a rare complication even after the intraneural administration of local anesthetics into the ScN (9–15, 22), perhaps because severe neural damage is the result of the interplay between multiple associated risk factors (6, 13, 19). However, the area of damaged nerve fibers with degeneration of the axons and myelin sheath affecting to one rabbit injected with bupivacaine could be indicative of the potential risks associated to intraneural injections in which the epineurium of an individual nerve is violated.

The needle type (sharp vs. blunt tipped) and its angle of insertion have been linked to the likelihood of inducing fascicular injury. In the current study, long-bevel needles were employed and nerves were punctured with an approximate angle of 30° in relation to the long axis of the ScN to ensure that the needle was secure in place at the selected sites of injection. Sharp needle tips increase the risk of fascicular injury, whereas a reduced puncture angle may decrease this risk (6, 11). Direct local anesthetic toxicity is recognized as a cause of nerve injury (6, 16–19, 33, 35), and a previous research found that bupivacaine caused more intense damage than lidocaine or ropivacaine to large-diameter nerve fibers of the ScN in rats injected intrafascicularly with these anesthetics (19). Surprisingly, the histological exams did not reveal significant differences in the histopathological scores between groups injected with saline or with bupivacaine in our research.

Limitations of this study were the short observational period considered after the injections as neurologic dysfunctions may occur weeks after the block, as well as the inability to perform electrophysiological studies in our laboratory. Nevertheless, in a previous work, the duration of the electrophysiological impairment after intraneural administration of local anesthetic in the ScN in humans was similar to that obtained after carrying out a conventional extraneural block (36).

The ScN has a unique anatomical structure (20–23, 27), which may explain the low vulnerability to structural damage and nerve dysfunction observed here after deliberate intraneural injections. Results from this research should not be extrapolated to other peripheral nerves nor to other species, as important differences in nerve microanatomy may produce different clinical outcomes. Future research should be carried out to elucidate the potential long-term clinical effects of these injections in the rabbit.

In summary, consequences of intraneural injections remain unclear, but these injections produced histological signs of mild perineural inflammation in most cases. The architecture of the nerves was well-maintained in 31/32 rabbits without changes in the integrity of the epineurium, perineurium, or fascicles at 7 days. Changes in nerve echogenicity and size observed after injections were not associated with clinically evident deficits in motor, proprioceptive, or sensorial function. Despite the lack of severe nerve disruption observed in most rabbits, the evidence of a focal area of damaged nerve fibers in one rabbit injected intrafascicularly with bupivacaine confirms that intrafascicular injections should be avoided as they may increase the risk of nerve damage.

## Data Availability Statement

The datasets generated for this study are available to any qualified researcher on request to the corresponding author.

## Ethics Statement

The animal study was reviewed and approved by University of Murcia Ethics Committee (approval number 218/2016).

## Author Contributions

AA, FL, and EB conceived and designed the study. AA, FL, EB, JM, and MS analyzed and interpreted the data. FG and JS-C analyzed the histopathological samples. FL and EB wrote the draft manuscript. All authors revised and discussed the manuscript, read, and approved the final version of the manuscript for publication.

### Conflict of Interest

The authors declare that the research was conducted in the absence of any commercial or financial relationships that could be construed as a potential conflict of interest.
